# Patient Health Record Systems Scope and Functionalities: Literature Review and Future Directions

**DOI:** 10.2196/jmir.8073

**Published:** 2017-11-15

**Authors:** Lina Bouayad, Anna Ialynytchev, Balaji Padmanabhan

**Affiliations:** ^1^ Department of Information Systems and Business Analytics Florida International University Miami, FL United States; ^2^ Health Services Research and Development Service, Center of Innovation on Disability and Rehabilitation Research Tampa, FL United States; ^3^ Department of Information Systems and Decision Sciences University of South Florida Tampa, FL United States

**Keywords:** personal health record systems, health records, personal, electronic health records, data analytics, medical informatics, patient-centered care, review, health platforms, multiorganizational systems, ultralarge systems

## Abstract

**Background:**

A new generation of user-centric information systems is emerging in health care as patient health record (PHR) systems. These systems create a platform supporting the new vision of health services that empowers patients and enables patient-provider communication, with the goal of improving health outcomes and reducing costs. This evolution has generated new sets of data and capabilities, providing opportunities and challenges at the user, system, and industry levels.

**Objective:**

The objective of our study was to assess PHR data types and functionalities through a review of the literature to inform the health care informatics community, and to provide recommendations for PHR design, research, and practice.

**Methods:**

We conducted a review of the literature to assess PHR data types and functionalities. We searched PubMed, Embase, and MEDLINE databases from 1966 to 2015 for studies of PHRs, resulting in 1822 articles, from which we selected a total of 106 articles for a detailed review of PHR data content.

**Results:**

We present several key findings related to the scope and functionalities in PHR systems. We also present a functional taxonomy and chronological analysis of PHR data types and functionalities, to improve understanding and provide insights for future directions. Functional taxonomy analysis of the extracted data revealed the presence of new PHR data sources such as tracking devices and data types such as time-series data. Chronological data analysis showed an evolution of PHR system functionalities over time, from simple data access to data modification and, more recently, automated assessment, prediction, and recommendation.

**Conclusions:**

Efforts are needed to improve (1) PHR data quality through patient-centered user interface design and standardized patient-generated data guidelines, (2) data integrity through consolidation of various types and sources, (3) PHR functionality through application of new data analytics methods, and (4) metrics to evaluate clinical outcomes associated with automated PHR system use, and costs associated with PHR data storage and analytics.

## Introduction

The idea of patient health records (PHRs) emerged in the early 1970s [1,2] with the goal of increasing patient engagement and empowerment, which in turn was intended to enable continuity of care, error reduction [3], treatment choice, and patient-provider partnership building [1,2].

An extension of traditional electronic health records (EHRs), PHRs created a patient-centric platform supporting the new vision of health services that enables patient-provider information sharing and collaboration, with the goal of improving health outcomes and reducing costs. In recent decades, great strides have been made toward achieving these far-reaching goals in research and practice. Through the implementation in the United States of the Health Information Technology for Economic and Clinical Health (HITECH) Act passed in 2009, the use of PHR data is becoming more commonplace [4]. As defined by the program, the initial stage of meaningful use encourages providers to integrate technology into medical practice, making vast amounts of patient data available electronically. Later stages of the program focus on empowering patients by providing them with online access to their heath data.

The use of PHRs has grown since the rise of mobile computing and advancement of patients’ technical aptitude. As an extension of EHRs, PHRs have been developed to enable patients to manage their own health care. These records include (1) EHR-transmitted data such as laboratory results and summary of care, and (2) patient-generated data such as symptoms. The amount of overlap in terms of data and functionalities between the EHR and PHR depends on the type of implementation: tethered, interconnected, or stand-alone [5]. Functionalities available through the PHR are intended to be used by patients, rather than by providers, and include appointment scheduling, prescription refill, and secure messaging [6]. The newly developed PHRs created a complementary source of clinical data such as patient-reported outcomes [7-9], physician ratings [10], medication adherence [11], and social support [12,13], and they allow for new data analytics techniques to detect, measure, and predict health-related outcomes. The United States has been a leader in the field of PHR data analytics. One reason for the growth of health care analytics in the United States is the incentivization of such research through federal initiatives to deliver patient-centered care and quality-driven payment models [14,15]. The Partnership for the Future of Medicare [15] states that innovative methods, such as email consultations and self-monitoring, must be used to achieve individualized, effective care. Additionally, Medicare strives to make health care data more readily available and accessible, including quality and performance metrics. Taken together, these initiatives support health care data collection and utilization in the United States, making PHR analytics more feasible. However, the full potential of PHR cannot be realized until we have a better understanding of PHR data content, formats, and sources.

Tremendous amounts of patient data are now available through PHR systems. With patients’ permission, these data, along with the application of advanced data mining and machine learning, can provide significant new opportunities in research. For instance, models in areas such as disease prediction, patient risk assessment, and early symptom detection can now be improved, leading to major advances in health outcomes and cost optimization. However, along with new opportunities provided by PHR systems come data and user-related challenges. Data-related issues such as quality, privacy, and security pertain to collection, safe storage, and processing of large quantities of patient data from distributed information systems. Also, patients previously excluded from access to such systems may lack the expertise to understand the data [16].

This review assessed the scope of data and functionalities in PHR systems with the goal of understanding how these affect research on health information systems. The platforms today lack a global standard and vary widely in terms of functionalities, goals, privacy issues, and legal frameworks. Hence, looking at the evolution of PHR data elements through a literature review of US studies, we also investigated opportunities and challenges associated with this emerging platform. While our review and implications are US centric, many of the broader research ideas have emerged from global applications.

## Methods

We conducted a review of US literature published from 1950 through 2015 to assess the scope and functionalities available through the PHR, along with associated data elements, formats, and sources. We summarized the results and classified the data content through functional categorization and chronological analysis, and identified gaps in the literature. Based on our findings, we present recommendations for health information systems research.

### Eligibility Criteria

In this review, we defined PHR as an electronic record designed for patients to self-manage care [6]. Thus, we focused on data that were either entered by or transmitted to the patient to enable self-care management, regardless of PHR type or brand.

We considered US studies from 1950 through 2015. We limited our search to US-based studies because of variation in ontologies and legal and privacy frameworks across countries. Because we were interested in specific data content available in the PHR, rather than patients’ extent of system use, we excluded articles focusing on PHR adoption. Furthermore, we excluded articles containing data intended to be used only by health care providers, and that not to be viewed by patients. For example, articles reporting on physician use of patients’ hormone levels to assess risk factors and clinical outcomes were excluded from the analysis because this information was not intended to be used or viewed by patients. Finally, after reviewing the body of articles selected based on title and abstract, we excluded articles that focused on general concepts and did not mention specific data elements present in the PHR.

### Data Sources and Search Strategy

**Figure 1:**
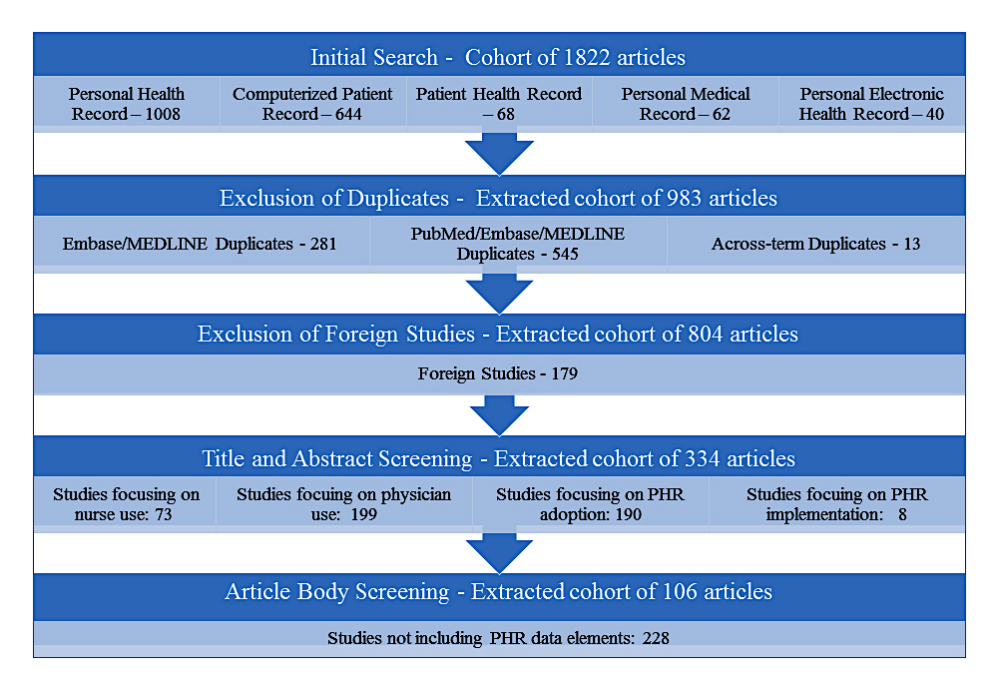
Literature review results. PHR: patient health record.

To conduct our review, and using used PubMed’s Medical Subject Headings (MeSH) database as our starting point, we identified 5 search phrases referring to the PHR: (1) personal health record, (2) personal medical record, (3) patient health record, (4) computerized patient record, and (5) personal electronic health record. A search of eligible US studies on PubMed from 1950 to 2015 and on Embase and MEDLINE from 1966 to 2015 using the previously defined phrases resulted in 1822 articles (Figure 1). The search results comprised articles containing any of the search phrases in all fields including titles and abstracts.

Title and abstract screening based on the inclusion and exclusion criteria by 2 reviewers (authors LB and AI) resulted in consideration of 334 articles. Data elements, associated data sources, and analytics techniques were described. The reviewers met after screening every 20 articles to compare results and adjudicate. Consensus was reached regarding (1) the final list of articles to be considered for full-text screening and (2) information extracted from the selected articles.

The full body screening resulted in a total of 106 articles used for data element extraction. Whenever available, reviewers LB and AI recorded the following information in an Excel 2010 (Microsoft Corporation) spreadsheet from each article reviewed in this study: (1) title, (2) author(s), (3) year of publication, (4) PHR data element(s) (ie, data collected by or shared via the PHR), (5) data type(s) (character, number, string, etc), (6) platform(s) (website, app, etc), (7) data storage (Excel database, Oracle, etc), (8) data entry (manual or electronic), (9) source, (10) receiver, (11) details regarding patient use, (12) barriers and issues, and (13) benefits.

### Data Categorization

A list of all data elements extracted from the 106 selected articles was further grouped by the reviewers into major data categories. The data categories were based on a taxonomy created in a PHR systematic review published in 2011 by Archer et al [6], which served as a foundation for this work. Categories found in our review but not included in Archer et al’s review were identified and validated by a group of clinical informatics experts.

We categorized the PHR data and refined them after consultation with an informal focus group of clinicians. In cases where different terms referred to the same data element (eg, medications, pills, and drugs), we chose 1 of the terms and grouped all synonymous data elements together under this term. Metadata pertaining to PHR functionalities were extracted from the articles and categorized based on content. For instance, articles mentioning the PHR reminder functionality were listed as references for data elements such as appointment reminders and prescription reminders, and were categorized under scheduling and treatments, respectively, as opposed to grouping all reminders under an umbrella “reminders” category. Additionally, some of the PHR data elements could have been included in different categories, depending on the user’s perspective. For example, the data elements described as prevention adherence could be viewed by the patient as part of a prevention plan but perceived by the provider as compliance with recommended health procedures and activities. We refined and ordered data categories listed in the results table based on their typical sequence of patient health care delivery. For example, scheduling data were listed before treatment data, which were listed before outcomes.

### Functional Taxonomy and Chronological Analysis

Following PHR data extraction and categorization, we performed a cross-categorical analysis of the data by percentage, source, and format. Additionally, we completed a longitudinal analysis of the time of first mention of the data element in the literature.

## Results

### Extraction Results

The literature review identified 13 major categories of PHR (Multimedia Appendix 1 [17-117]). At least one data element was included within each of the main categories, and details on the data elements and their corresponding references are provided. In addition to the data elements previously reported in Archer et al’s systematic review, this research identified 22 new data elements. Additionally, we distinguished 3 data elements from Archer et al’s review in the more recent PHR literature and separated them into more than one data element.

Patient data elements reported in the literature are available in Multimedia Appendix 1.

The comparable data elements identified in both reviews were personal information, problem lists, surgical history (procedures, hospitalizations), medical history (family history), provider information (provider list), allergies, home monitoring data, medical history, psychographics (social history, lifestyle), immunizations, prescription medications, and notes.

The data elements not previously reported in Archer et al and that we identified in this research were (1) genetic data, (2) preferences, (3) PHR settings, (4) facility information, (5) personalized search results, (6) visit preparation information, (7) compliance, (8) medical equipment and supplies, (9) self-treatment, (10) treatment plan, (11) outcomes, (12) patient-provider message, (13) incentive programs data, (14) patient health education material, (15) trainings, (16) personalized health advice, (17) environmental information, (18) assessment information, (19) personal health goals, (20) health care cost management, (21) insurance data, and (22) health status.

In our research, we were also able to separate Archer et al’s preventive health recommendations into (1) preventive care and (2) prevention adherence. We broke examinations and diagnoses down into (1) vital signs and anthropometric data, (2) physiological information, and (3) diagnosis. We further distinguished laboratory tests and appointments as (1) results, (2) imaging, and (3) appointments.

### Functional Taxonomy and Chronological Analysis Results

We grouped PHR data elements by source, format, and time of first mention. Analysis of data elements mentioned in the literature allowed for description of information available for analytics use. This analysis also revealed the capabilities available to patients through PHR systems.

#### Patient Health Record Systems Data—Scope

**Figure 2:**
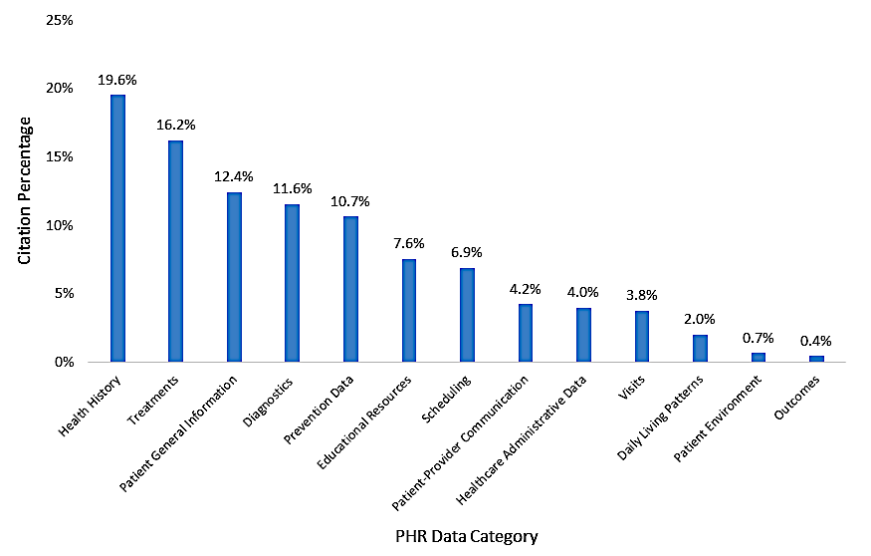
Patient health record (PHR) data category by citation percentage.

The bar graph in Figure 2 displays the frequency of data elements described in the articles we reviewed. To obtain the percentages, we divided the total number of citations for each of the major data categories by the total number of citations for all major data categories combined. Figure 2 shows a wide range in the frequency of data categories described in the literature, with health history being the most frequently occurring data category, accounting for 88 out of 450 (19.6%) total citations, and outcomes being the least frequently mentioned, accounting for 2 out of 450 (0.4%) citations.

The 4 most frequently occurring data elements (health history, treatments, patient general information, and diagnostics) accounted for 269 out of 450 (59.8%) total citations and were typically added to PHRs through extraction from the patients’ EHR.

In addition to data elements extracted from the EHR, a significant amount of data, such as information about medication adherence and self-care, is entered by patients. However, we found PHR-entered data less frequently in our review, representing about 27% of the 450 total citations: 34 (7.6%) citations related to educational resources, 31 (6.9%) citations related to scheduling, 19 (4.2%) citations related to communication, 17 (3.8%) citations related to visits, 9 (2.0%) citations related to daily living patterns, 3 (0.7%) citations related to patient environment, and 2 (0.4%) citations related to outcomes. These likely reflect new functionalities provided to patients through their PHR during our review period. Administrative data accounted for 18 (4.0%) of total citations and consisted of information on health care cost management and insurance data. Health care cost management included information on admissions and discharges and on health spending. Insurance information, on the other hand, provided patients with information such as insurance claims, benefits, copays, and reimbursement.

Data available in the PHR were generated by a multitude of devices, and were entered by different parties (ie, patients and providers) through various platforms (Table 1). We found that data elements related to the patient-provider encounter, such as patient general information, diagnostics, psychosocial status, treatments, visits, and outcomes data, were generally extracted from the EHR. More recent data elements were entered through patient portals (such as educational resources and patient environment data), or transmitted by sensors and tracking devices (such as daily living patterns).

The variety of PHR platforms led to the generation of different data formats (Table 1). Newly generated patient data were not limited to plain text and numbers in structured tables. Electronic messages, for example, were composed of text and metadata describing the time of transmission and the identity of sending and receiving parties. Templated documents and forms were used for standard reports such as legal documents, care plans, and insurance reports [46]. Images, also prevalent in PHRs today, were used by patients and providers to capture, store, and transmit health data, such as radiology results (2-dimensional x-rays, 3-dimensional computed tomography scans, positron emission tomography scans, magnetic resonance imaging scans, 4-dimensional beating heart) [84], signs and symptoms (wound images) [91], camera uploads [31], health trends (growth charts) [46], mood graphs [37], blood sugar graphs[99], laboratory flow sheets [31], and legal documentation (power of attorney for children and adolescents) [22]. Audio and video were used to capture phone call content [46] and record visits [46]. Newer data formats generated by patient tools and mobile apps included Google Maps for facility information and Google Calendar entries associated with appointment scheduling [31].

**Table 1:** Patient health record data: common formats and sources.

Main data source and data category		Main data type						
		Text	Number	Image	Video	Voice	Time series	GIS^a^ or map
**Electronic health record**								
	Patient general information	X	X					
	Diagnostics	X	X					
	Psychosocial status	X	X					
	Treatments	X	X					
	Visits	X	X					
	Outcomes	X	X					
**Patient portal or mobile device**								
	Educational resources	X	X	X	X	X		
	Scheduling	X	X					X
	Patient environment	X	X					
	Patient-provider communication	X	X	X				
	Patient-provider communication	X	X	X				
**Administrative record**								
	Administrative data	X	X					
**Sensors or tracking devices**								
	Prevention data	X	X	X	X	X	X	
	Daily living patterns	X	X	X	X	X	X	

^a^GIS: geographic information system.

**Figure 3:**
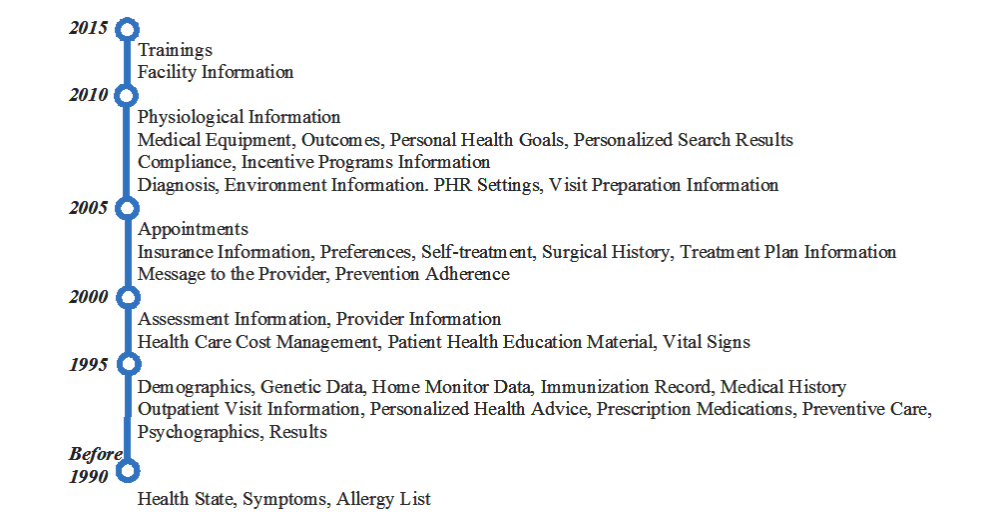
Patient health record (PHR) data elements by year of first mention.

#### Patient Health Record Systems Data—Evolution Over Time

Next, we analyzed the data elements extracted by the year of first mention (Figure 3). In the early 1990s, PHR data elements mentioned in the literature pertained to researchers’ and practitioners’ visions of potential future systems. These included general patient data, such as demographics, and medical encounter information, such as visit summary.

After initial uses of PHR systems in the early 2000s, new data elements such as appointments, preferences, and system settings emerged. More recently, PHR data included reminders (eg, appointment reminders [51,99,101], medication reminders [93,110,114], screening and laboratory work reminders [42,46,110], immunization reminders [29,30,55,57,82,90], preventive care reminders [21,59,60], and health maintenance reminders [82]), in addition to alerts [22,76,77,99], identification of personal health goals [19,24,38-40,43,72,74], and disease prevention [76,77,99,110,115]. Tracking and monitoring data via e-journals [82] and diaries [50] also became available.

Today, PHR data are generated through different tools and devices. Tracking devices, now transmitting time-series PHR data, are used to monitor patients’ vital signs, such as blood pressure and glucose level (biomonitoring devices) [74,99], and to detect abnormal events, such as alerts from implantable cardioverter defibrillators [117].

#### Patient Health Record Systems Functionalities—Scope

PHR data were mainly used to provide added functionalities to patients. The provider search results [20,22,47,49,64], for example, helped patients locate health care providers and health-related services. Similar functionalities enabled patients to obtain health advice from support groups. Other functionalities assisted patients with preparing for medical encounters through visit preparation questionnaires [24,46,66,70-72]. Functionalities such as incentive programs [43,56,66,73,74] empowered patients through self-health monitoring. Finally, a unique PHR data category discovered in our review, environmental information [36,50,56,67], captured community health concerns and environmental domains, which can be linked to functionalities such as assessment of environment-related risk factors and recommendations for preventive care.

#### Patient Health Record Functionality Evolution Over Time

**Figure 4:**
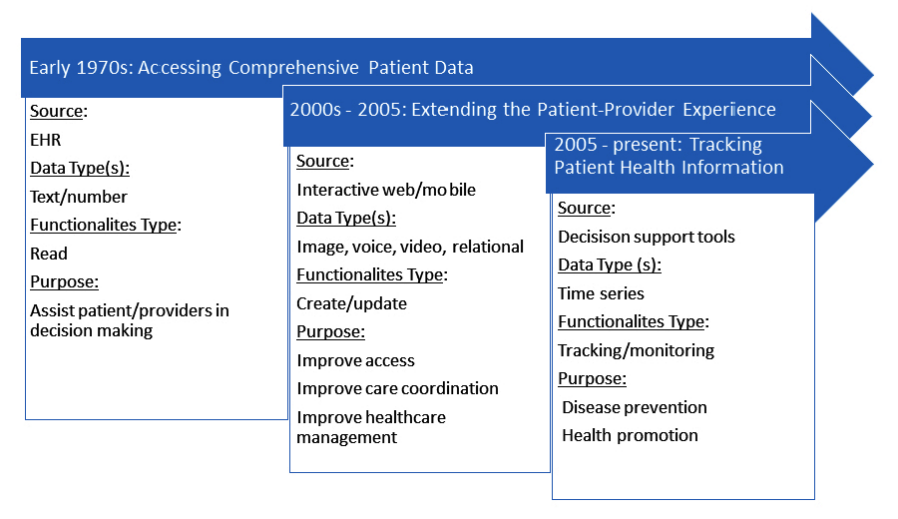
Patient health record functionality evolution over time, showing the most common sources, data types, and functionalities found in the review. EHR: electronic health record.

Description of the data extracted revealed which functionalities were available to the patient through the PHR and indicated an interesting evolution of PHR functionalities (Figure 4).

The evolution of PHR data elements over time (Figure 4) illustrates the general inclination in the early stages toward providing the patient with access to health information regarding their medical encounter.

Even though the giving patients access to their own health data was initiated in the 1970s, PHR systems were not widely used until the early 2000s. Because of the infancy of PHR systems, research in this domain has focused on system adoption and how it relates to patient satisfaction. Only limited research is available on how to leverage PHR data to improve health outcomes.

Starting in 2005, data elements reported in the literature indicate a shift toward a more interactive view of the PHR system and the introduction of several new attributes and functionalities. Patient PHR settings, including security and privacy preferences, became more prevalent. The most significant development of this time period of PHR evolution was the interaction and engagement of the patient with the system. Functionalities such as patient-provider secure messaging and appointment scheduling were becoming more common.

More recently, the PHR system has seen a greater inclusion of patient tracking and monitoring functionalities as daily reported data from patients and caregivers become more prevalent. Albeit rare, PHR systems also increasingly allow for cost measurement and management.

## Discussion

### Implications and Future Directions—PHR Data

Overall, the results indicate an increasing focus in the literature on newer types and sources of data, as well as on providing patients with access to their health data. Yet some of these may be progressing so rapidly that important related issues are somewhat neglected. Few studies, for instance, have examined the impact of user interface design on patients’ understanding of data and system use. Issues associated with the use of PHRs are mainly related to patients’ understanding of the underlying information presented. Problems related to understanding of health data may lead to stress and anxiety [63], which could outweigh the potential benefits of data access. Hence, research is needed in the area of data visualization and representation models specifically targeted for patient use. Examples of such models available in the literature are the what-if analysis, [99] brief intervention [109], and traffic-light feedback system [74]. These methods indicate the risks associated with specific health activities, along with related outcomes and recommended interventions. The traffic-light feedback system, for example, provides patients with an effective visualization tool to track their progress toward attainment of blood pressure goals.

In addition, more research is needed to investigate and improve the quality of patient-entered data. Today, more than 35,000 mobile health apps are available for the iOS and Android operating systems, generating large amounts of data [118]. Data are also increasingly entered through patient forums and portals. While new platforms allow the generation and availability of large data volumes, the wide variety of levels of expertise could lead to reliability and validity issues. Patient-entered data have been shown to be reliable for simple measures such as demographics and symptoms, but less reliable when they pertain to reporting more complicated measures such as laboratory values [5]. One method for improving accuracy could be to provide patients with standardized measures and guidelines for entering their own data, but even that needs to be part of a broader strategy to verify accuracy of data through triangulation from multiple sources.

As the variety of PHR data sources increases, special care is needed for data curation [119] and harmonization [120]. Processes need to be established to produce usable patient-reported data that can be used for research [121]. Standards need to be developed to improve interoperability between different components of the new PHR systems [122]. Data integration methods, such as entity stream mining [123], might be required to cross-reference patient data generated by different tools and devices.

In the coming years, PHR systems will create many data-related challenges, such as quality, heterogeneity, openness, security, scalability, and transparency. Abundant patient data might also trigger information overload. While potentially beneficial for improving health outcomes, streaming patient data can amount to very large volumes, creating new data quality, storage, and analysis issues. All of these challenges open doors for valuable research in health information systems.

The large amounts of data generated by sensors and devices might also require storage and analysis on the cloud [118], potentially increasing storage and analysis costs. Sharing patient data between networks may also create a risk of personal health information disclosure [124], generating additional costs for preserving patient privacy and security. This could also necessitate stronger methods for patient data protection beyond today’s practice, which opens up yet another important avenue for health informatics research.

### Implications and Future Directions—PHR Functionalities

Overall, PHR data evolution indicates a general trend toward greater patient engagement and health tracking. Moving forward, a continuation of these trends will lead to accumulation of vast amounts of rich data. If patients provide permission, research on PHR data can pave the way for patient-centered care.

The design of patient-centered decision support systems that use a combination of comprehensive individual patient information and aggregate data (collections of patient records) to provide personalized patient recommendations will be a significant area of research.

While past literature has listed patient-provider messaging as an important communication tool for patients and providers, secure message content may potentially provide a valuable patient data source for analysis. Based on their reported intended use, patient secure messages may contain information regarding health-related concerns such as new symptoms and adverse events. Among other possibilities, information retrieved from secure messages could, therefore, be used in research to identify treatment side effects and build patient risk models. However, it is important to keep in mind that terminology used by patients is likely to differ from terminology used by providers. Hence, natural language processing models traditionally used to extract patient information from provider notes may need to be adapted to fit the patient context.

Recently developed and highly effective deep learning algorithms could also be used to extract, search, sort, and analyze information from the tremendous amounts of image, voice, and video data [125] available in the PHR. Other new techniques might be needed to analyze relational data, such as from Google Maps and Google Calendars.

Also, current methods used to store, extract, and analyze EHR data are not adequate for analysis of large volumes of time-series data. Nonrelational databases might be needed to store tracking information. Stream learning algorithms [126] would also need to be applied to extract meaningful information from the terabytes of streaming data analyzed.

As patient-centered decision support systems are being implemented, it is important to ensure the validity of the generated output. Misclassification errors can be dangerous in this domain. Patient systems, which are embedded in mobile devices, need to be evaluated and approved by medical experts. Data transmitted from different sources can potentially be leveraged by providers to improve patient and population health outcomes. However, accurate measures are still needed to assess and improve the performance of such systems. In addition, these metrics need to account for biases present in patient-generated data. Prior research indicated that PHR systems are mostly used by patients who are typically more sick. Those are patients with comorbidities, such as cancer survivors [127]. Therefore, findings and models generated from analyzing these data might not be generalizable to other patient populations.

The new health care vision in the United States is characterized by automation and collaboration, creating the need for adaptation by all actors in the industry. Empowered patients today have the opportunity to leverage PHR systems data and functionalities. This, however, requires some level of technical expertise for system access and interaction, and medical knowledge in order to understand and interpret the medical information presented. Similarly, medical providers now have to learn and adopt new technologies in order to report medical data and communicate with patients. As a major actor in the health care industry, insurance companies also need to adapt to the new industry environment. Insurance firms today need to assess the value of virtual medical encounters and automated care, and process new types of patient data such as secure messages. Adaptation methods by all health industry players are yet to be assessed and optimized.

### Limitations

A limitation of this study is its focus on PHR data reported in the literature. The evolution of PHRs as described in this study might not necessarily reflect the state of the practice. More research is therefore needed to extract and evaluate PHR scope and the functionalities of the various PHR systems available in practice. Also, as mentioned above, this study focused on US studies, thereby limiting the scope of our analysis. Research comparing PHR systems in the United States with those used in other countries would help improve future data uses.

### Conclusions

Digital health platforms have changed drastically in recent years. The introduction of distributed PHR systems enabled a shift toward more personalized and increasingly automated health care. The multiuser nature of PHR systems also facilitated patient-to-provider and patient-to-patient information sharing. Yet these changes generated opportunities and challenges at the user, system, and industry levels. Our assessment here of the state of the patient digital infrastructure serves as a valuable foundation for future research. Research implications identified also offer ways to significantly advance health information systems research. Identifying available PHR data also facilitates the development of intelligent health systems. Although primarily aimed at health information systems researchers, implications listed in this study can be further extended to health practitioners, insurance providers, and policy makers.

## Appendix

Multimedia Appendix 1 Patient data elements reported in the literature.

## Abbreviations

EHR: electronic health record
HITECH: Health Information Technology for Economic and Clinical Health
MeSH: Medical Subject Headings
PHR: patient health record
