# Multimedia Appendix Correction: Patient Health Record Systems Scope and Functionalities: Literature Review and Future Directions

**DOI:** 10.2196/15796

**Published:** 2019-09-05

**Authors:** Lina Bouayad, Anna Ialynytchev, Balaji Padmanabhan

**Affiliations:** 1 Department of Information Systems and Business Analytics Florida International University Miami, FL United States; 2 Health Services Research and Development Service, Center of Innovation on Disability and Rehabilitation Research Tampa, FL United States; 3 Department of Information Systems and Decision Sciences University of South Florida Tampa, FL United States

The authors of “Patient Health Record Systems Scope and Functionalities: Literature Review and Future Directions” (J Med Internet Res 2017;19(11):e388) have replaced Multimedia Appendix 1 with a new file. Because of formatting issues, the original appendix was missing data elements in the table. Published here is a new version of Multimedia Appendix 1 with all data elements.

The corrections will appear in the online version of the paper on the JMIR website on September 5, 2019, together with the publication of this correction notice. Because this was made after submission to PubMed, PubMed Central, and other full-text repositories, the corrected article also has been resubmitted to those repositories.

## Appendix

Multimedia Appendix 1 Patient data elements reported in the literature.

