# Supplementary material for: Patient Health Record Systems Scope and Functionalities: Literature Review and Future Directions
Source: J Med Internet Res. 2017 Nov 15;19(11):e388. doi: 10.2196/jmir.8073 (PMC5707430; doi:10.2196/jmir.8073)
Supplement: Multimedia Appendix 1 [file jmir_v19i11e388_app1.pdf]

	Data Elements*	References
Patient General Information	**Personal Information**<br>Name, photo, date of birth, age, gender, current location, contact information, occupation, number of family members, economic history	[17–33]
	**Psychographics**<br>Leisure, travel history, living conditions, psychosocial information (e.g. social contact, interpersonal relations, caregiving, neglect, abuse), social information history, patient empowerment; patient satisfaction, socializing, Observations of Daily Living, mood, alcohol use, tobacco use, sexual behavior, Tobacco cessation	[18,21,23,27,29–31,34–43]
	**Genetic Data**	[18]
	**Preferences**<br>Hospital preferences, pharmacy preferences, emergency contact information, caregiver information, advance directive, living will, power of attorney	[3,22,27,29,31,37,44–48]
	**PHR Settings**<br>Reminder preferences; activation, profile; security and privacy settings registration and authentication (log-in) information; secure message opt-in; protection settings	[22,25,29,31,34,38,46,49–53]
Scheduling	**Appointments**<br>schedule date and time, appointment reminders	[19,21,25,26,29,30,42,46,47, 54–63]
	**Facility Information**<br>Facility contact information	[29]
	**Personalized Search Results**<br>Healthcare providers, health-related services	[20,22,47,49,64]
	**Provider Information**<br>Example: provider (pediatrician, dental) name, photograph, number, address	[3,19,20,22,24,27,31,32,37,48,63,65]
Visits	**Outpatient Visit Information**<br>Visit summary (clinical summary), provider progress notes	[18,21–23,26,28,32,43,46,47,58,63,63,66–69]
	**Visit Preparation Information**<br>Items to discuss during the visit (examples: care plan using patient chart data, patient goals, referrals, self-care, and patients' responses to pre-visit questionnaires)	[24,46,66,70–72]
Diagnostics	**Vital Signs & Anthropometric Data**<br>Examples: blood pressure (BP), blood glucose, body weight, body mass index, pediatric measures (head circumference and developmental milestones, growth chart)	[22,23,25,29,30,32,37,39,40, 46,47,55,59–62,68–70,73–76]
	**Physiological Information**<br>Electrocardiograms, device readings (infra-red and biosensors, video cameras)	[36,37,76–78]
	**Results**<br>Lab (chemistry and hematology), imaging, microbiology, pathology, and screenings	[3,18,19,21–23,25,26,28,30,31,37,39,42,46,47,50,56,59–63,66,68–70,73,75,79–89]
History of present illness	**Allergies**<br>Examples: allergy record by date, allergic reaction, allergy diagnosis	[2,3,17,21–24,26,27,29,31,33,38,44,48,63,67,69,83,90]
	**Diagnosis**	[19,21,38,42,47,48,50,91,92]
	**Health State**<br>Health conditions trends, health status, physical condition, mental health condition, medical health record, medical records review, oral health, eye records	[2,3,18,21–23,28–30,33,35,44,48–50,53,57,58,63,70,77,93,94]

	**Medical History**<br>Medical history, family health history	[17,18,22,25,27–29,31,42,44,47,48,50,56,57,61,65,66,68,69,71,73,80,83,90,95–99]
	**Surgical History**<br>Emergency health record (care procedures), hospitalizations	[3,17,22,24,27,31,38,47,56,78]
	**Problem List**<br>Pre-existing symptoms, new symptoms	[2,17,18,21–24,26–28,31,33,36,38,60,61,63,67,69,71,77,92]
**Treatments**	**Compliance**<br>Medication monitoring data (e.g. medication possession ratio), medication adherence outcomes, perceived barriers, medication reminders	[51,66,75,100–102]
	**Medical Equipment/Supplies**<br>Technology support needs (ventilator, monitor, surgical site care)	[22]
	**Prescription Medications**<br>Active list (dose, schedule), interaction record and checks, prescription photograph, history, refill request, prescription feedback, medication reconciliation	[3,18,20–27,29–31,33,34,36,38–40,42,44,46–52,54–57,60–63,66–69,71,75,80,82–84,88,90,91,93,95,102–109]
	**Self-Treatment**<br>Example: over-the-counter medications, herbals and other supplements	[3,17,29,44,55,82]
	**Treatment Plan**<br>Treatment plan description, treatment plan change, specialist referral	[3,17,21,27,36,42,43,46–48,56,60,71,90,92,110]
**Outcomes**	**Outcomes**<br>Patient health outcomes, adverse reactions	[22,35,62]
**Patient Provider Communication**	**Patient-Provider Message**<br>Secure messages (content: non-urgent health-related information, appointment requests, prescription renewal, health-related concerns)	[21,25,29,30,35,39–41,52,54–56,61–63,67–70,73,80,88,91,93,95,111]
**Educational Resources**	**Incentive Programs Data**<br>Certificates of accomplishments (for completing online educational quizzes)	[43,56,66,73,74]
	**Patient Health Education Material**<br>Instructions and educational material regarding health conditions, immunizations, over-the-counter medications, contraindicated self-care activities. clinical trial data	[21,23,25,29,37–40,43,46,47,54,56,57,59,61,64,66,68,69,73,78,81,83,88,93,112,113]
	**Trainings**<br>Self-management trainings, patient coaching	[114]
	**Personalized Health Advice**<br>Advice from health support groups (support from patients with similar health conditions), personalized advice/tips	[18,25,46,55,59,68,100]

**Patient Environment**	**Environmental Information**<br>Community health concerns; environmental domain (example: income, sanitation, residence, neighborhood-workspace safety)	[36,50,56,67]
**Prevention Data**	**Assessment Information**<br>Screening exams, risk/health assessment questions and feedback, routine screening, screening/lab work reminders, potential risk factors, risk profile, personal disease risk	[3,29,31,32,42,43,46,50,81,94,110,111,115]
	**Immunizations**<br>Status, eligibility, due dates, availability, counter-indications, family immunization history, information about disease risk minimization, immunization reminders	[3,18,21,26,29,31–33,38,46–48,50,63,68,69,83,84,112,115]
	**Personal Health Goals**<br>Desired and planned values (blood sugar, cholesterol/BP), progress towards goals	[19,24,38–40,43,72,74]
	**Prevention Adherence**<br>Compliance with guideline-recommended services, alerts (e.g. high BP, weight loss)	[69,76,77,100,111,113,116]
	**Preventative Care**<br>Supplements (e.g. herbal), self-care activities, daily aspirin, preventive care reminders, health maintenance reminders	[18,30,55,57,64,72,79,83,91]
**Daily Living Patterns**	**Home Monitoring Data**<br>Nutrition, diet, meals, food intake, exercise, physical activity, estimated calories burnt, health diaries, health maintenance schedule and activities, symptom monitoring, health tracking	[3,17,18,23,31,36,43,62,65,72,100,117,118]
**Healthcare Administrative Data**	**Health care cost management**<br>Examples: admissions/discharges, health spending, billing data	[23,25,26,42,46,47,56,63]
	**Insurance Data**<br>Examples: insurance claims, benefits, copay, reimbursement	[3,26,27,30,31,38,44,47,48,79,80,91]

Table 1: Patient data elements reported in the literature

*Data description includes a list of items retrieved from the articles reviewed and is not comprehensive
